# Engineering of a borneol dehydrogenase from *P. putida* for the enzymatic resolution of camphor

**DOI:** 10.1007/s00253-021-11239-5

**Published:** 2021-04-12

**Authors:** Michael Hofer, Julia Diener, Benjamin Begander, Robert Kourist, Volker Sieber

**Affiliations:** 1 Fraunhofer Institute for Interfacial Engineering and Biotechnology IGB, Schulgasse 11a, 94315 Straubing, Germany; 2 Chair of Chemistry of Biogenic Resources, Campus Straubing for Biotechnology and Sustainability, Technical University of Munich, Schulgasse 16, 94315 Straubing, Germany; 3 Institute of Molecular Biotechnology, Graz University of Technology, Petersgasse 14, 8010 Graz, Austria

**Keywords:** Borneol dehydrogenase, Enzymatic resolution, Camphor, Racemate

## Abstract

**Abstract:**

Several thousand different terpenoid structures are known so far, and many of them are interesting for applications as pharmaceuticals, flavors, fragrances, biofuels, insecticides, or fine chemical intermediates. One prominent example is camphor, which has been utilized since ancient times in medical applications. Especially (−)-camphor is gaining more and more interest for pharmaceutical applications. Hence, a commercial reliable source is needed. The natural sources for (−)-camphor are limited, and the oxidation of precious (−)-borneol would be too costly. Hence, synthesis of (−)-camphor from renewable alpha-pinene would be an inexpensive alternative. As the currently used route for the conversion of alpha-pinene to camphor produces a mixture of both enantiomers, preferably catalytic methods for the separation of this racemate are demanded to yield enantiopure camphor. Enzymatic kinetic resolution is a sustainable way to solve this challenge but requires suitable enzymes. In this study, the first borneol dehydrogenase from *Pseudomonas* sp. ATCC 17453, capable of catalyzing the stereoselective reduction of camphor, was examined. By using a targeted enzyme engineering approach, enantioselective enzyme variants were created with *E*-values > 100. The best variant was used for the enzymatic kinetic resolution of camphor racemate, yielding 79% of (−)-camphor with an *ee* of > 99%.

**Key points:**

*• Characterization of a novel borneol dehydrogenase (BDH) from P. putida.*

*• Development of enantioselective BDH variants for the reduction of camphor.*

*• Enzymatic kinetic resolution of camphor with borneol dehydrogenase.*

**Graphical abstract:**

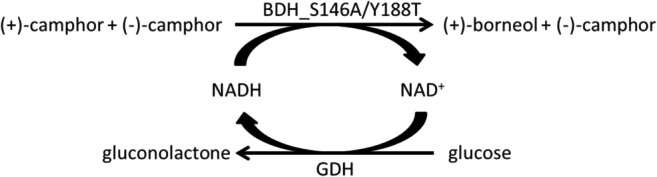

## Introduction

**Fig. 1:**
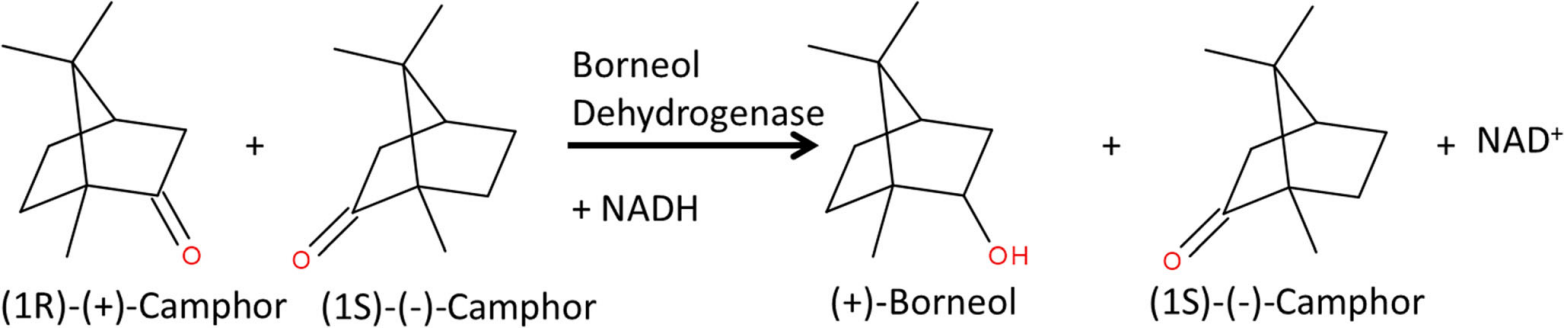
Enzymatic kinetic resolution of camphor racemate using an enantioselective borneol dehydrogenase

Terpenes are hydrocarbons classified by the number of isoprene units (C_5_H_8_), ranging from hemiterpenes (one isoprene unit) to polyterpenes (more than eight isoprene units), and among natural compounds, they form one of the largest groups. Monoterpenes (C_10_H_16_) are dimers of isoprene and occur in acyclic, monocyclic, bicyclic, and tricyclic forms. Monoterpenes are typically derivatized to containing oxygen or nitrogen atoms and are then referred to as monoterpenoid*s*. Camphor is a bicyclic monoterpenoid containing oxygen. In nature, it is synthesized in plants (Banthorpe et al. 1972) and degraded by microorganisms such as *P. putida* (Bradshaw et al. 1959). Plants synthesize (+)- as well as (−)-camphor, but the most common and economically available enantiomer is (+)-camphor produced from the camphor tree, whereas (−)-camphor is available by the oxidation of (−)-borneol but only at high costs. In addition, camphor as racemic mixture can be synthesized cost-efficiently from alpha-pinene, which is a side product from the pulp and paper industry (Zhen-dong and Liang-wu 2009). The demand for (−)-camphor for the synthesis of pharmaceuticals is constantly growing (Xu et al. 2005; Selescu et al. 2013; Sherkheli et al. 2013); therefore, a simple and efficient approach for the separation of camphor racemate is highly desirable. Enzymatic kinetic resolution of racemic mixtures is a common way to solve this challenge (Breuer et al. 2004; Verho and Backvall 2015). For example, alcohol dehydrogenases (ADH) have been widely used for the oxidation of alcohols as well as for their reduction. Currently, no camphor-specific alcohol dehydrogenase is available; however, several borneol dehydrogenases (BDH) are known. *Salvia officinalis* harbors a (+)-borneol selective dehydrogenase, whereas *Tanacetum vulgare* harbors a (−)-borneol selective dehydrogenase (Dehal and Croteau 1987; Drienovská et al. 2020), whereas a non-selective alcohol dehydrogenase from *Artemisia annua* is described converting (+)- and (−)-borneol (Polichuk et al. 2010) as well as a (+)-borneol selective variant (Tian et al. 2015). Interestingly, the most active BDH enzymes to date are not from plant but have a bacterial origin and are the non-stereoselective borneol dehydrogenases from *Pseudomonas* sp. strain TCU-HL1 (Tsang et al. 2016). Besides BDH enzymes, only a tropinone reductase from *Cochlearia officialis* showed activity in oxidation of (−)-borneol as well as reduction of (+)-camphor (Reinhardt et al. 2014). However, none of these enzymes has shown activity or selectivity in the reduction of (+/−)-camphor that would suffice for the synthesis of optically pure (−)-camphor. In this study, the first borneol dehydrogenase capable of reducing camphor could be identified. Unfortunately, the wild-type enzyme has only a very low enantioselectivity. By modeling the enzyme structure, crucial residues for enantioselectivity were identified and used as targets for site-directed mutagenesis, resulting in several improved variants with an *E*-value > 100 and with high remaining enzyme activity. The best enzyme variant was used for the catalytic separation of a camphor racemate (Fig. 1).

## Materials and methods

### Materials

Glucose dehydrogenase from *Pseudomonas* sp. was purchased from Sigma-Aldrich (UK).

#### Bacterial strains and growth conditions

*E. coli* DH10B was used as host for cloning procedures and *E. coli* BL21(DE3) for protein production. The strains were grown in LB liquid media or on LB agar plates at 37 °C for biomass production. Media were supplemented with 100 μg/ml kanamycin. Borneol dehydrogenase production was induced upon addition of IPTG (final concentration 1 mM) at an OD_600_ of 0.6, and cultivation was continued for 16 h at 16 °C. Cells were collected by centrifugation (4000 × *g*, 10 min, 4 °C). *Pseudomonas* sp. ATCC 17453 was used for cloning of the borneol dehydrogenase gene. Cells were grown on LB liquid media or LB agar plates at 30 °C.

#### Cloning

*Pseudomonas* sp. ATCC17453 was grown on LB agar containing 100 μg/ml ampicillin at 30 °C. A single clone was picked and transferred into 10 μl aqua dest. and used as DNA template. The PCR conditions for amplification of the *bdh* gene (GenBank: AB771747.1) were 5 min at 98 °C for DNA denaturing, followed by 25 cycles of denaturing at 98 °C for 10 s, primer annealing at 61 °C for 20 s, and strand extension at 72 °C for 30 s. A last incubation step at 72 °C for 10 min finished the reaction using phusion DNA polymerase. The following pair of oligonucleotides was used for the reaction: 5-GATCCCATGGATGAAACCGCTAGCAGG-3 and 5-GATAGCGGCCGCTCAACCAGACAATCGATTG-3. For the creation of His-tagged variant, following primers were used: 5-TTGGTCTCGGATGCACCACCACCACCACCACAAACCGCTAGCAGGTAAAAG-3 and 5-GATAGCGGCCGCTCAACCAGACAATCGATTG-3. The PCR products were digested with *Nco*I or *Bsa*I and *Not*I and cloned into pET28a digested with the same restriction enzymes. All constructs were subsequently sequenced by Eurofins Genomics Germany GmbH.

#### Enzyme modeling and substrate docking

The three-dimensional structural models of *Pseudomonas* sp. ATCC 17453 borneol dehydrogenase were generated with the program SWISS-MODEL (Waterhouse et al. 2018), using the crystal structure of 3-alpha, 20 beta-hydroxysteroid dehydrogenases (PDB: 2HSD) as template (Ghosh et al. 1994). The protein and the substrate docking files were prepared using MGLtools (Morris et al. 2009). The substrate was docked into the models with AutoDock Vina (Trott and Olson 2010), based on the binding mode of NAD^+^ in the template and the position of the catalytic residues in the models. The exhaustiveness was set to 8, otherwise default parameters were used. The structure figures were prepared with the software program PyMOL v 2.4.

#### Enzyme engineering

The wild-type enzyme was engineered by site-directed mutagenesis at positions Q96, H98, E145, S146, N154, and Y188. The primers for mutagenesis were designed as described (Edelheit et al. 2009). PCR conditions were 2 min at 95 °C for DNA denaturing, followed by 16 cycles of denaturing at 95 °C for 45 s, primer annealing at 55 °C for 30 s, and strand extension at 72 °C for 12 min. A last incubation step at 72 °C for 10 min finished the reaction using Pfu DNA polymerase (Promega GmbH, Germany). Afterwards, 1 μl DpnI was added to the reaction mixture and incubated for 2 h at 37 °C, before ligation and transformation of *E. coli* DH10B were performed. All amino acids were mutated to amino acids of different properties or size creating seven focused libraries (Table 1). In the second mutation round, the best variants from round one were combined.

**Table 1:** Selected amino acid changes for the site-directed mutagenesis

Q96: R, F, Y, W, K, E, N, L, M	
H98: F, Y, W, Q, R, K, E, L, M, N	
E145: M, L, N, I, S, H, K, R, Q, F, Y, W, G	
S146: G, A, V, I, L, N, D, E, T	
N154: L, I, M, E, S, H, K, W, Y, F, Q, R	
Y188: W, H, R, Q, F, K, E, L, M, N	

#### Protein purification

All purification steps were performed at 4 °C. The cell pellet was resuspended in buffer A (50 mM potassium phosphate buffer pH 8.0, 20 mM imidazole, and 0.5 M sodium chloride). Then cells were disrupted in a French press at 1350 bar (TS 0.75, Constant Systems, UK). After centrifugation for 30 min at 30.000 × *g*, the soluble fraction containing BDH was passed through 0.2 μm sterile filter, before loading on a 1 ml HisTrap FF crude column (GE Life Sciences, USA). The column was previously equilibrated with buffer A. The column was washed with 50 mM potassium phosphate buffer pH 7.5, containing 50 mM imidazole and 0.5 M sodium chloride, and BDH was eluted with 50 mM potassium phosphate buffer pH 7.5, containing 300 mM imidazole and 0.5 M sodium chloride. The BDH fractions were pooled and desalted using a HiPrep 26/10 Desalting column (GE Life Sciences, USA) and 50 mM potassium phosphate buffer pH 7.5. The protein stock could be stored for at least 1 week at 4 °C with only minor losses of activity. For the determination of optimum pH in regard to activity, the enzyme was desalted in 50 mM potassium phosphate buffer ranging from pH 6.5 to 9 or 50 mM citrate-phosphate buffer ranging from pH 4 to 6.5. Molecular absorption coefficient and molecular weight values of the proteins were determined via Expasy-Protparam (Gasteiger et al. 2005). Protein concentrations were calculated according to the Beer-Lambert law, after measuring the absorbance at 280 nm.

#### Enzyme analysis

The oxidation reaction mixture contained 30 μg/ml enzyme, 50 mM Tris-HCl at pH 7.5, 1 mM NAD^+^ and 1 mM substrate at 30 °C. The reaction was started by the addition of enzyme. The reduction reaction mixture contained 500 μg/ml enzyme, 50 mM citrate-phosphate buffer at pH 5.0, 1 mM NADH, and 1 mM substrate at 30 °C for the enzyme analysis. The screening of engineered enzyme variants was done in 50 mM Tris-HCl at pH 7.5, 1 mM NADH with 12.5 mM formate, and 1 mg/ml formate dehydrogenase. The reaction was started by the addition of BDH enzyme. For determination of pH optimum, the enzyme was desalted in the corresponding buffer and activity measured as described. The temperature optimum was determined by incubation of the assay solutions before and during the reaction at the corresponding temperature. Kinetic analysis was done at optimal pH and temperature values. The *K*_*m*_ and *k*_*cat*_ of NAD^+^ were determined in the presence of 1 mM substrate and 0.01, 0.02, 0.04, 0.08, 0.1, 0.2, 0.4, 0.6, 0.8, and 1 mM cofactor. The *K*_*m*_ and *k*_*cat*_ for (+)-borneol and (−)-borneol were determined in the presence of 1 mM NAD^+^ and 0.02, 0.04, 0.08, 0.1, 0.2, 0.4, 0.6, 0.8, 1, and 2 mM substrate. For data analysis, the enzyme kinetics module of SigmaPlot v13.0 was used.

#### Calculation of enantioselectivity

The enantiomeric ratio was calculated by Eq. (1). The conversion of the reaction was calculated according to Eq. (2).

1 $$ E=\ln\ \left(\left({\mathrm{ee}}_p\ast \left(1-{ee}_s\right)\right)/\left({ee}_p+{ee}_s\right)\right)/\ln \left(\left({ee}_p\ast \left(1+{ee}_s\right)\right)/\left({ee}_pp+{ee}_s\right)\right) $$

2 $$ Conversion={ee}_s/\left({ee}_s+{ee}_p\right) $$

*ee*_*s*_ and *ee*_*p*_ are the *ee* values of the remaining substrate and the formed product

#### Deracemization

For the enzymatic kinetic resolution of camphor, 500 μg/ml of BDH was added to 50 mM citrate-phosphate buffer at pH 5.0, containing 0.05 mM NADH, 12.5 mM glucose, 17 U/ml glucose dehydrogenase, and 1 mM camphor racemate. The mixture was incubated at 30 °C under shaking for 2 h. Samples were taken after 0, 15, 30, 45, 60, 75, 90, 105, 120, 135, and 150 min and analyzed by GC as described.

#### GC analysis

Assay products were analyzed after product extraction from assays with equivalent amounts of ethyl acetate containing 1 mM hexylbenzene as internal standard. GC analysis was performed with a Shimadzu GCMS model 2010 Q Plus connected to a MS detector (Single Quad). Chromatographic separation was achieved on a FS-HYDRODEX β-6TBD capillary column (25 m × 0.25 mm); 1.0 μl of the sample was injected at 250 °C (Split-Splitless injector). The oven temperature was initially maintained at 60 °C for 8 min, then raised to 150 °C at 2.0 °C per min, and then raised to 200 °C at 45 °C per min and finally held for 2 min. The column flow (carrier gas: helium) was 1.9 ml/min. The products were identified by comparison of the retention times and mass spectra with authentic standards.

## Results

### Cloning, protein expression, and purification

**Fig. 2:**
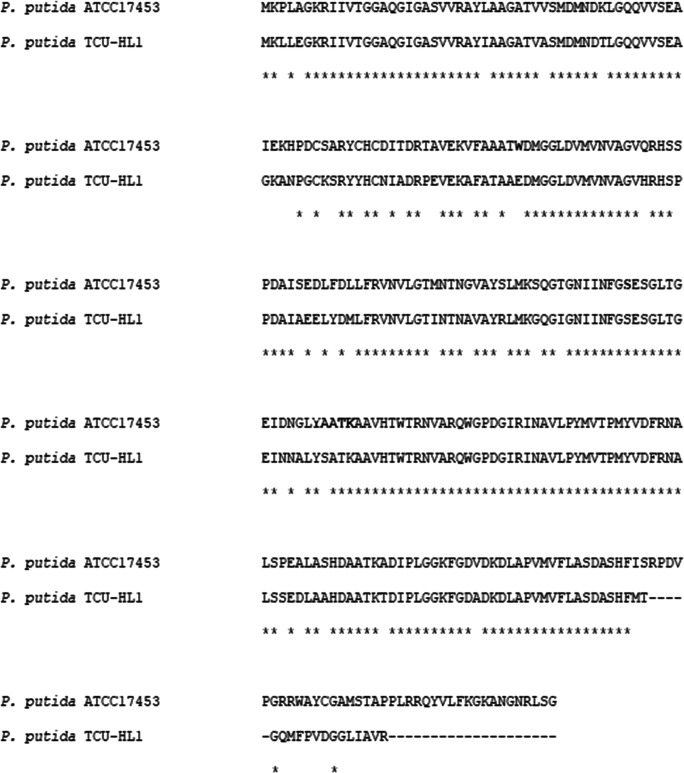
Sequence alignment of the cloned BDH gene from *Pseudomonas* sp. ATCC17453 with BDH from *Pseudomonas* sp. TCU-HL1 (Tsang et al. 2016) using ClustalW 1.7. Identical amino acids are marked with *. Active site amino acids are in bold

*Pseudomonas* sp. ATCC17453 contains a borneol dehydrogenase on the CAM plasmid, which is part of the camphor degradation pathway (Bradshaw et al. 1959; Hartline and Gunsalus 1971). The cloned *bdh* gene encodes a polypeptide chain of 260 amino acids with a predicted mass of 27.5 kDa and a pI of 5.35. A NAD(H)-binding motif G_13_XXXGXG_19_ and a catalytic motif Y_157_XXXK_161_ with a S_144_ residue show that the enzyme belongs to the class of short-chain dehydrogenases (Kavanagh et al. 2008). In *Pseudomonas* sp. TCU-HL1, the *bdh* gene has also a length of 260 amino acid residues and a similarity of 84% on protein-level with BDH from *Pseudomonas* sp. ATCC17453 (Tsang et al. 2016) (Fig. 2). After cloning into the expression vector pET28a with an N-terminal His-Tag, the protein could be expressed in *E. coli* in the soluble fraction and purified by nickel affinity chromatography, resulting in a single protein band with the expected size of 31 kDa (data not shown).

### Protein characterization

**Fig. 3:**
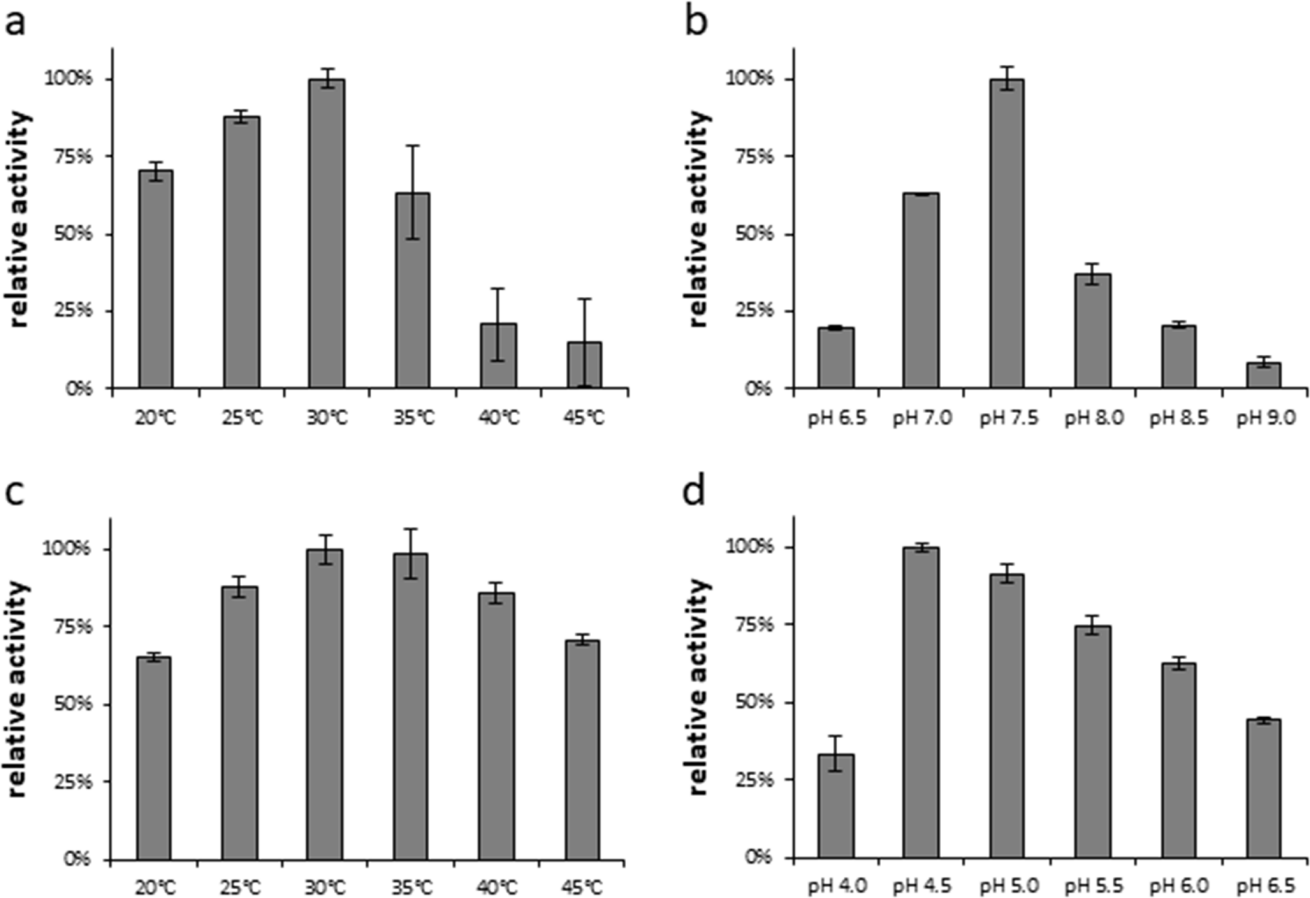
Characterization of BDH from *Pseudomonas* sp. ATCC17453. (**a** + **b**) Temperature and pH optimum for the oxidation of (±)-borneol. (**c** + **d**) Temperature and pH optimum for the reduction of camphor

As shown in Fig. 3, BDH from *Pseudomonas* sp. ATCC17453 has its optimum at pH 7.5 and an optimal reaction temperature of 30 °C for the oxidation of borneol. The pH optimum for BDH from *Pseudomonas* sp. TCU-HL1 with an optimum at pH 8.5 differs slightly. The analysis of the enzyme kinetic data revealed a Michaelis Menten behavior with apparent *K*_*m*_ values for (+)-borneol of 0.095 ± 0.004 mM and 0.115 ± 0.002 mM for (−)-borneol and 0.06 ± 0.005 mM for NAD. The corresponding *k*_*cat*_ values for (+)-borneol and (−)-borneol were 0.89 and 0.97 s^−1^, respectively. This data is similar to the kinetic analysis of BDH from *Pseudomonas* sp. TCU-HL1 with *K*_*m*_ values for (+)-borneol 0.2 ± 0.01 mM and 0.16 ± 0.01 mM for (−)-borneol and *k*_*cat*_ values for (+)-borneol 0.75 and 0.53 s^−1^ (Tsang et al. 2016). BDH showed no preference for either one of the two borneol isomers (*E* = 1.7). Besides (+)- and (−)-borneol, also (±)-isoborneol was accepted as substrate, whereas isopropanol, cyclopentanol, cyclohexanol, 1,2-butandiol, (±)-2-butanol, L-carveol, and DL-menthol were not accepted.

### Reduction of camphor

In contrast to all other described BDH enzymes so far (Polichuk et al. 2010; Sarker et al. 2012; Tian et al. 2015; Tsang et al. 2016; Drienovská et al. 2020; Khine et al. 2020), BDH from *Pseudomonas* sp. ATC17453 is able to catalyze the reduction of camphor to borneol with a maximal conversion rate at 30 °C and a pH optimum at 4.5 (Fig. 3). Since the protein was precipitating at pH 4.5, all assays were performed at pH 5.0. A detailed kinetic analysis was not possible since substrate concentrations above the solubility level of camphor (1.25 g/l) were necessary. The specific activity of the enzyme with (+)-camphor was 0.2 U/mg and with (−)-camphor 0.18 U/mg at a substrate concentration of 1 mM.

### Molecular modeling, docking, and protein engineering

**Fig. 4:**
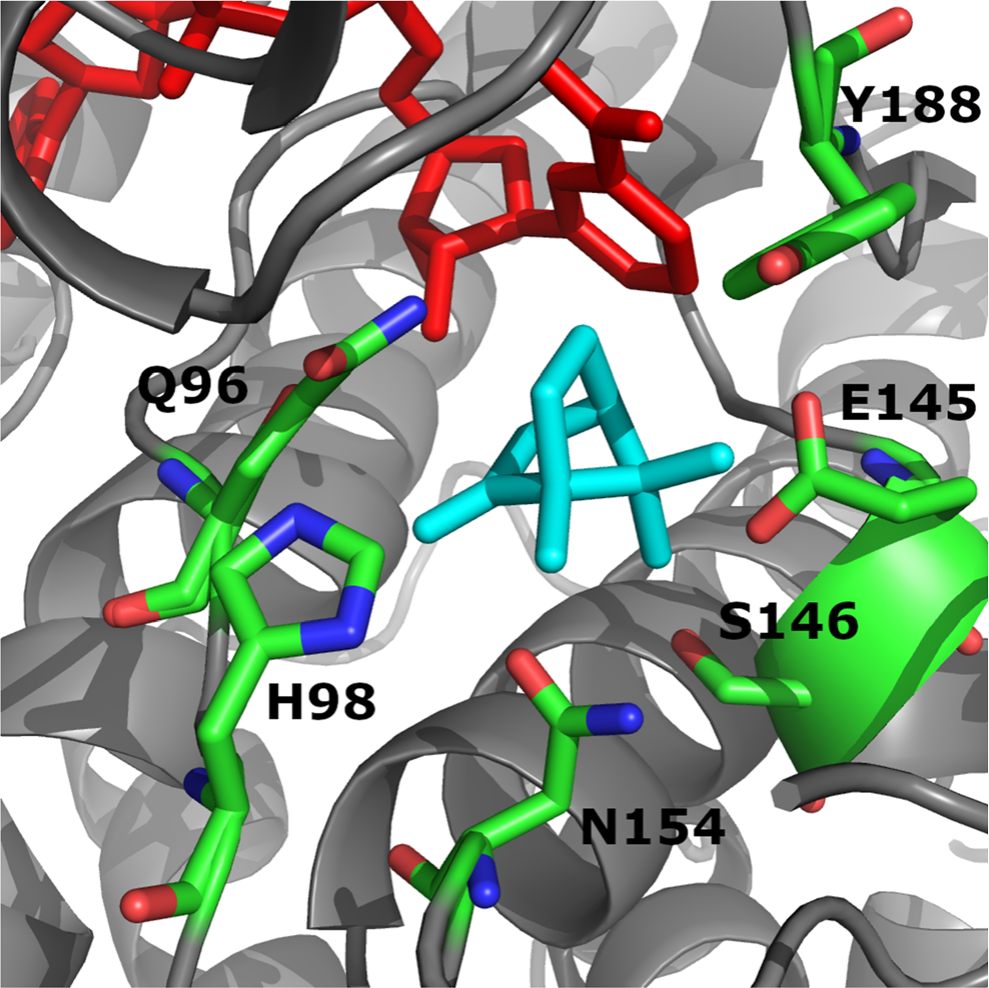
Model of the substrate binding pocket of BDH from *Pseudomonas* sp. ATCC17453. NADH is shown in red, camphor is shown in cyan, and amino acids chosen for mutagenesis are shown in green

The amino acid sequence of BDH from *Pseudomonas* sp. ATCC17453 shows 28% identity and 37% sequence similarity to the previously crystallized short-chain dehydrogenase 3*α*,20*β*-hydroxy-steroid dehydrogenase from Streptomyces hydrogenans (EC 1.1.1.53; Ghosh et al. 1994), which is the most similar crystallized enzyme so far. Therefore, 3*α*,20*β*-hydroxy-steroid dehydrogenase was chosen as template to build the BDH homology model. In accordance with the sequence data, BDH prefers NADH as electron donor in the kinetic assay and was therefore used for modeling. The calculated model containing the cofactor as well as substrate provided a framework for the choice of amino acid residues for mutagenesis (Fig. 4). In the substrate binding pocket, the side chains of Q96, H98, E145, S146, N154, and Y188 are in close range to the substrate (5 Å) and not part of the active side. Accordingly, these residues were postulated to affect enzyme–substrate interaction, thus determining the substrate selectivity to yield (+)-camphor-specific variants. Therefore, these amino acids were selected for site-directed mutagenesis.

### Enzyme engineering for improved enantioselectivity

BDH from *Pseudomonas* sp. ATCC17453 is not enantioselective for camphor (Table 2). Therefore, site-directed mutagenesis was used to create focused libraries at six selected positions in the substrate binding pocket as described. None of the variants at position N154 showed any effect on the enzyme’s enantioselectivity, whereas variants from libraries Q96, H98, and E145 only showed a minor improvement of camphor selectivity (*E* (+) < 4) (data not shown). Eleven variants from libraries E145, S146, and Y188 showed a significant increase in (+)-camphor selectivity (*E* (+) > 4) (Table 2). Nine of them had *E*-values ranging from 6 to 12.1. The best variants BDH_Y188T and BDH_Y188A showed synthetically useful *E*-values (*E* > 100). With the prospect to improve the conversion rate of (+)-camphor while retaining the high enantioselectivity, all variants of the first screening round with a conversion rate > 10% were combined with Y188T and Y188A, and the resulting double mutants were also screened. The combination of the mutations resulted in several highly enantioselective variants (Table 3). Although only the double mutants BDH_S146A/Y188T and BDH_S146A/Y188A resulted in higher conversion rates (40% and 39%) compared to the single mutants. The kinetic parameters of both mutants show an increased reaction velocity compared to the wild type for the oxidative reaction from (+)-borneol to camphor (Table 4).

**Table 2:** Kinetic resolution of racemic camphor catalyzed by improved mutants of borneol dehydrogenase from *Pseudomonas* sp. ATCC17453. The enantiomeric ratio E was calculated using Eq. (2)

BDH variant	*ee*_*s*_	*ee*_*p*_	Conversion	E (+)
Wild type	24%	9%	27%	1.7
BDH_S146A	8%	71%	11%	6.3
BDH_E145G	6%	71%	8%	6.3
BDH_E145H	4%	71%	5%	6.0
BDH_E145M	27%	68%	29%	6.9
BDH_E145L	30%	67%	31%	6.6
BDH_E145S	59%	59%	50%	7.0
BDH_Y188L	4%	71%	6%	6.2
BDH_Y188I	9%	74%	11%	7.3
BDH_Y188V	17%	82%	17%	12.1
BDH_Y188T	28%	98%	22%	> 100
BDH_Y188A	29%	99%	23%	> 100

**Table 3:** Kinetic resolution of camphor catalyzed by improved double mutants of borneol dehydrogenase from *Pseudomonas* sp. ATCC17453

BDH variant	*ee*_*s*_	*ee*_*p*_	Conversion	E (+)
Wild type	24%	9%	27%	1.7
BDH_E145M/Y188T	11%	97%	10%	72.6
BDH_E145M/Y188A	2%	94%	2%	30.5
BDH_E145L/Y188T	19%	100%	16%	> 100
BDH_E145L/Y188A	12%	99%	11%	> 100
BDH_E145S/Y188T	5%	96%	5%	50.0
BDH_E145S/Y188A	20%	100%	16%	> 100
BDH_S146A/Y188T	65%	97%	40%	> 100
BDH_S146A/Y188A	64%	99%	39%	> 100

**Table 4:** Kinetic analysis of wild type as well as enantioselective borneol dehydrogenase variants from *Pseudomonas* sp. ATCC17453

BDH variant	*K*_*m*_ (−)-borneol [mm]	*K*_*m*_ (+)-borneol [mm]	*k*_*cat*_ (−)-borneol [s^−1^]	*k*_*cat*_ (+)-borneol [s^−1^]
Wild type	0.11	0.09	0.97	0.89
BDH_S146A/Y188T	0.73	0.46	0.13	3.32
BDH_S146A/Y188A	0.34	0.28	0.07	1.51

### Enzymatic kinetic resolution of camphor

**Fig. 5:**
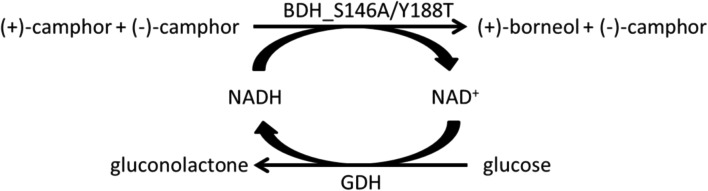
Enzymatic kinetic resolution of camphor. GDH catalyzes the conversion of glucose to gluconolactone while NAD^+^ is reduced. BDH catalyzes the conversion of (+)-camphor to (+)-borneol while NADH is oxidized to NAD^+^

**Fig. 6:**
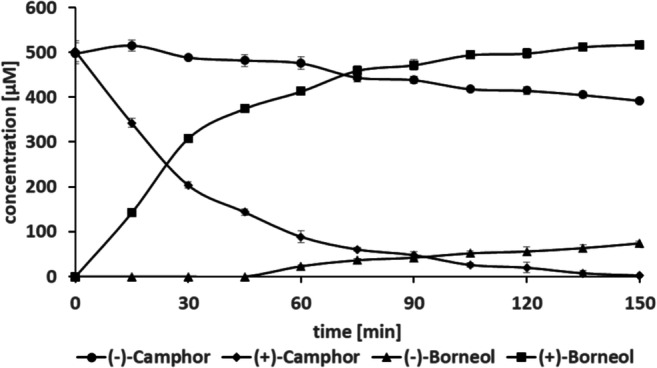
Time course of the enzymatic kinetic resolution of camphor

For the targeted deracemization of camphor, BDH was coupled with glucose dehydrogenase (GDH) for cofactor recycling (Fig. 5), due to the inactivity of formate dehydrogenase under optimal BDH conditions. GDH catalyzes the oxidation of glucose to gluconolactone and reduces NAD^+^ to NADH, which is subsequently used by BDH for camphor racemate reduction to yield (+)-borneol and (−)-camphor. To investigate the suitability of GDH for the cascade, first, a steady-state analysis of the enzyme under the optimal reaction conditions for the recombinant BDH (30 °C, pH 5.0) was done. The in silico analysis of the enzyme kinetics data revealed a Michaelis Menten behavior with apparent *K*_*m*_ values for glucose 3.6 mM ± 0.35 and *k*_*cat*_ 1.3 s^−1^. Next, GDH and BDH were combined in a ratio that reflects similar activities under optimal reaction parameters to promote an optimal hydride flow. After 2.5 h, > 99% of (+)-camphor was converted to (+)-borneol with an *ee*_*P*_ of 75.1%, while the process yielded 79% of the target compound (−)-camphor with an *ee*_*S*_ of > 99% (Fig. 6). Reducing the incubation time increases the yield of (−)-camphor but at the expense of reduced *ee*_*S*_.

## Discussion

BDH from *Pseudomonas* sp. TCU-HL1 and the BDH from *Pseudomonas* sp. ATCC17453 have a similarity of 84% with only 41 differing amino acids. Point mutations are well known for influencing protein solubility (Smialowski et al. 2007; Tian et al. 2010; Klesmith et al. 2017), which might be the reason for the good solubility of BDH from *Pseudomonas* sp. ATCC17453 in contrast to the low solubility of BDH from *Pseudomonas* sp. TCU-HL1. The activity of BDH from *Pseudomonas* sp. ATCC17453 is much higher than the known BDH from plant (Croteau et al. 1978; Dehal and Croteau 1987; Polichuk et al. 2010; Tian et al. 2015; Fu et al. 2016; Drienovská et al. 2020) with *k*_*cat*_ values for (+)-borneol and (−)-borneol of 0.89 and 0.97 s^−1^. It lies in the same range as BDH from *Pseudomonas* sp. TCU-HL1 with 0.75 and 0.53 s^−1^ for (+)-borneol and (−)-borneol. Only recently, a novel bacterial BDH enzyme was discovered with the highest activity among all known BDH enzymes so far, with *k*_*cat*_ values for (+)-borneol and (−)-borneol of 75.0 and 11.3 s^−1^ (Khine et al. 2020). Interestingly, this enzyme is also the first BDH enzyme, which prefers (+)-borneol as substrate. Whereas the wild-type BDH from *Pseudomonas* sp. ATCC17453 shows no enantioselectivity, it is the first BDH enzyme capable of catalyzing the reverse reaction from camphor to borneol. None of the other studies regarding borneol dehydrogenase has analyzed the reverse reaction (Croteau et al. 1978; Dehal and Croteau 1987; Polichuk et al. 2010; Tian et al. 2015; Fu et al. 2016; Drienovská et al. 2020; Khine et al. 2020) nor could detect any reductive activity of the enzyme so far (Sarker et al. 2012). A tropinone reductase-like short-chain reductase was reported, able to convert pure (+)-camphor and (−)-borneol with a *k*_*cat*_ of 0.06 and 0.09, respectively (Reinhardt et al. 2014). Recently, an alcohol dehydrogenase from *Salvia rosmarinus* showed also low activity in the reduction of racemic camphor (Chánique et al. 2021). Due to the low activity, these enzymes are unsuitable for the desired kinetic resolution of camphor. By using BDH from *Pseudomonas* sp. ATCC17453, an enzymatic kinetic resolution of camphor racemate to yield pure (−)-camphor is realizable. Since the wild type shows virtually no enantioselectivity, enzyme engineering was applied to yield variants with outstanding selectivity. It has been shown that mutations close to the substrate binding site are more efficient in generating enantioselective enzyme variants (Morley and Kazlauskas 2005). By applying this strategy to the wild-type BDH, six positions close to the modeled substrate could be identified as targets for a semi-rational engineering approach (Q96, H98, E145, S146, N154, and Y188). The selected positions are not directly involved in cofactor binding or the catalytic mechanism. A common approach is to use site-directed saturation mutagenesis or CASTing (combinatorial active-site saturation test) as efficient engineering strategy to reduce the screening space (Reetz 2011). In this study, the screening space was further reduced, by a limited site-directed mutagenesis approach only generating 9–12 mutations at each site instead of 20 to reduce the screening effort. Through this approach, double mutants with high enantioselectivity and high activity were generated in just two mutation rounds. The best variant, BDH S146A/Y188T, has an *E*-value > 100 for (+)-camphor and is therefore useful for the enzymatic kinetic resolution of camphor. For an efficient process, the cofactor NADH has to be recycled to drive the equilibrium to (+)-borneol (Kroutil et al. 2004). Therefore, a coupled enzyme approach with glucose dehydrogenase and glucose as second substrate was established. This approach resulted in *ee*_*S*_ of > 99% with a yield of 79% of (−)-camphor. Although the enantioselectivity is quite good, the overall yield of (−)-camphor is not reaching the theoretical yield due to a low activity of the mutant enzyme with (−)-camphor. Further enzyme engineering has to be applied to realize an economical viable deracemization process.

## Data Availability

All data generated or analyzed during this study are included in this published article (and its supplementary information files).
